# Full-Length Transcriptome Sequencing Provides Insights into Flavonoid Biosynthesis in *Fritillaria hupehensis*

**DOI:** 10.3390/life11040287

**Published:** 2021-03-28

**Authors:** Kunyuan Guo, Jie Chen, Yan Niu, Xianming Lin

**Affiliations:** 1 Institute of Chinese Herbal Medicines, Hubei Academy of Agricultural Sciences, Enshi 445000, China; gky@hbzyc.com.cn; 2 Wuhan Benagen Tech Solutions Company Limited, Wuhan 430070, China; chenjie@benagen.com (J.C.); niuyan@benagen.com (Y.N.)

**Keywords:** third generation sequencing, medicinal plant, herbal medicine, genomic analysis

## Abstract

One of the most commonly utilized medicinal plants in China is *Fritillaria hupehensis* (Hsiao et K.C. Hsia). However, due to a lack of genomic resources, little is known about the biosynthesis of relevant compounds, particularly the flavonoid biosynthesis pathway. A PacBio RS II sequencing generated a total of 342,044 reads from the bulb, leaf, root, and stem, of which 316,438 were full-length (FL) non-redundant reads with an average length of 1365 bp and a N50 of 1888 bp. There were also 38,607 long non-coding RNAs and 7914 simple sequence repeats detected. To improve our understanding of processes implicated in regulating secondary metabolite biosynthesis in *F. hupehensis* tissues, we evaluated potential metabolic pathways. Overall, this study provides a repertoire of FL transcripts in *F. hupehensis* for the first time, and it will be a valuable resource for marker-assisted breeding and research into bioactive compounds for medicinal and pharmacological applications.

## 1. Introduction

*Fritillaria hupehensis* (Hsiao et K.C. Hsia) belongs to the *Liliaceae* family, and is a bulbous medical herb found in China, particularly in the southwestern parts of Hubei province [1,2]. The bulb is documented in Chinese Pharmacopeia as a major Chinese medicinal herb [1,3]. Fritillaria is the biopharmaceutical source of many drug compounds used in traditional Chinese medicine [4]. The bulbs are famed for important bioactive compounds such as glycosides, terpenoids, saponins, and steroidal alkaloids [1,3,5,6,7]. Nonetheless, the flower, leaf, root, and stem are also used in folk medicine for their therapeutic and curative properties [4,8,9]. Fritillaria has also been used as an antitussive, anti-asthmatic, and expectorant and as a treatment for hot-type bronchitis with dry cough and for heart diseases. Recently, the bulb was employed for the treatment of scrofulous inflammations and breast lumps and as a key constituent in herbal preparations for cancer treatment [4].

The abundant pharmacological and phytochemical profiles in Fritillaria species have made them the objects of diverse studies, and a broad variety of new steroid alkaloids have been recently isolated [7,10]. Understanding biosynthetic pathways is necessary for the efficient utilization of key phytochemicals present in *F. hupehensis* [5,9,11]. Comprehensive transcriptome studies on medicinal plants such as *Panax quinquefolius* (Linn.) [12,13,14], *Glycyrrhiza glabra* (Linn.) [1,15], and *Salvia miltiorrhiza* (Bunge.) [12] have been reported. In *Fritillaria cirrhosa* (D. Don)*,* earlier authors [16,17] investigated the biosynthetic pathways for steroidal alkaloids, which synthesize the main bioactive components responsible for the pharmacological properties. Zhao et al. [5] used RNA-seq for the identification of 113,865 unigenes involved in *F. cirrhosa* secondary metabolites biosynthesis. The methylerythritol phosphate (MEP) pathway was found to be the principal route to steroidal alkaloid biosynthesis in *F. cirrhosa*. About 105,522 unigenes were annotated for the first time from the full-length transcriptome profiling of *Panax quinquefolius* L. [5,13,18]. De novo transcriptome sequencing of *Astragalus membranaceus* (Fisch.) yielded 9732 unigenes and novel metabolic pathways [19]. The multi-component and multi-target mechanisms of secondary metabolites and their regulatory mechanisms have gained prominence in modern Chinese medicinal research [19,20,21]. Alkaloids, flavonoids, glycosides, organic acids, saponins, and steroids, and other secondary metabolites are important pharmaceutical and phytochemical enzymes involved in metabolic processes [11,21,22].

Transcriptome sequencing is an efficient method for identifying transcripts, discovering new genes, and determining which genes are expressed in plants. When compared with transcriptomes assembled using second-generation sequencing platforms, full-length (FL) transcripts sequencing made possible by third-generation sequencing improves transcriptome characterization accuracy [6,23,24]. Third-generation sequencing (PacBio RS II) has been utilized previously to study the genetic resources of medicinal plants [25,26]. The PacBio RS II is the world’s first commercially available third-generation DNA sequencer, comprising novel single-molecule real-time (SMRT) technology [27,28]. In comparison with second-generation sequencing platforms, PacBio RS II offers significantly longer read lengths, high consensus accuracy, low bias, and simultaneous epigenetic characterization [29]. PacBio RS II sequencing also improves mapping of sequencing data, correctly identifies intron–exon boundaries, alternatively spliced transcripts, transcription start and end sites, and precise strand orientation to single exons. As a result, it has become the preferred technology for genome and transcript sequencing [30,31,32,33]. For species without a reference genome, the FL transcriptome is the most advanced genomic resource available, and it can be used for functional genomics study and molecular breeding.

The PacBio RS II was used to sequence and characterize the FL transcriptome of *F. hupehensis* in this study. Unique genes were detected and important biosynthetic pathways were identified. The phylogenetic relationships of the flavonoid genes were particularly analyzed. To our best knowledge, this is the first FL transcriptome profiling report for *F. hupehensis* and can be used as a valuable resource for further functional study in *F. hupehensis*.

## 2. Materials and Methods

### 2.1. Plant Materials

*F. hupehensis* (Hsiao et K.C. Hsia) plants were cultivated for over three years in the Huazhong Chinese medicine botanical garden in Enshi City in 2017, Hubei Province, at an altitude of 1680 m. The bulbs, leaves, roots, and stems were harvested in the first, third, and fifth days after anthesis (DAA). Three biological replicates of each tissue from at least five plants with a consistent genetic background and growth rates were sampled and mixed. Following that, the samples were frozen in liquid nitrogen for RNA extraction and sequencing.

### 2.2. RNA Extraction, Preparation of PacBio SMRT Library, and Sequencing

Total RNA was extracted from the bulb, leaf, root, and stem samples (4 tissues × 3 biological repeats) of *F. hupehensis* using the Trizol RNA extraction kit (Invitrogen, Carlsbad, CA, USA). The NanoDrop 2000 spectrophotometer was used to check the RNA concentration and purity (Thermo Fisher Scientific, Wilmington, DC, USA). The extracted RNAs were combined to provide a full RNA. Using a magnetic d(T) bead binding procedure, mRNA was isolated from total RNA and transcribed to cDNA with the use of a Clontech SMARTer PCR cDNA Synthesis Kit (Clontech Laboratories, Inc., CA, USA). In selecting PCR products, the BluePippinTM Size Selection Method (Sage Science, Beverly, MA, USA) was used and fragments of 0.5–6 kbs were retained. Long-scale PCR was then used to enhance the cDNA. The cDNA ends were repaired and the sequence adapters linked to cDNA were ligated. Bell libraries for the SMRT templates were developed with cDNA and sequenced on the PacBio Sequel platform with P6-C4 chemistry, 10-h film times. The Gene Denovo Biotechnology Company (Guangzhou, China) performed all sequencing work. Raw reads were further filtered to attain clean reads by exclusion of adaptors, reads with more than 10% of unknown nucleotides, and poor quality reads. Clean reads, Q30, and GC content were computed.

### 2.3. Full-Length Transcriptome Profiling

The complete transcriptome analysis involved long sequence identification and isoform clustering to achieve a consistent sequence [34]. The sequences of the reads of insert (ROI) were removed from the original sequences. The cDNA primers and polyAs were filtered and grouped based on 3, 5, and A; long and non-FL sequences; and chimeric and non-chimeric sequences. The iterative isoform-clustering algorithm was used to cluster the entire isoform sequences and group the full sequences with equal sequences. There was a consistent sequence in each cluster. Finally, the results were polished using the Quiver algorithm.

### 2.4. Transcriptome Annotation

Raw reads in fastq format were processed using in-house Perl scripts. To obtain clean reads, we deleted from the raw data reads with adapters and polyNs and low-quality reads. At the same time, we calculated the clean reads, sequence duplication levels, Q20, Q30, and GC content. All downstream analyses were based on high quality clean data. All library/sample read1 files were combined into a large, left file (read1 files). The right files (read2 files) were also pooled into one large file right.fq. Transcriptome assembly was performed with trinity64 [35] on the left.fq and right.fq, min kmer cov set to 2 by default. Single gene functions were annotated based on Clusters of Orthologous Groups (COG) [36], Gene Ontology (GO) [37], Kyoto Encyclopedia of Genes and Genomes (KEGG) [38], Non-supervised Orthologous Group (eggNOG) [39], NCBI non-redundant protein sequence database (NR) [40] and Protein family (Pfam) [41,42].

### 2.5. Identification of Simple Sequence Repeats and Long Non-Coding RNAs 

The MicroSAtellite identification tool (MISA; http://pgrc.ipk-gatersleben.de/misa/; accessed on 22 November 2020), an online Perl script program, was used to identify simple sequence repeats (SSRs) in *F. hupehensis*. As a search criterion, the minimum unit size for tri- to hexa-nucleotide repeats was set at five, and the minimum unit size for di-nucleotide repeats was set at six. CPC [43], CNCI [44], Pfam [41,42], and CPAT [45] were used to identify the long non-coding RNAs (LncRNAs) in the transcriptome.

### 2.6. Identification of Flavonoid-related Genes and Phylogenetic Analysis

The corresponding flavonoid-associated genes with the highest similarity in *Solanum lycopersicum* (SL4.0) and *Arabidopsis thaliana* (TAIR9 genome release) were retrieved from NCBI using putative genes involved in the flavonoid biosynthetic pathway as seed sequences [46]. After confirming their conserved domains in the NCBI conserved domain database (https://www.ncbi.nlm.nih.gov/Structure/cdd/wrpsb.cgi; accessed on 22 November 2020), the protein sequences were pooled and aligned in MEGA 10.0 using the ClustalW alignment tool (MEGA; https://www.megasoftware.net/; accessed on 22 November 2020). The evolutionary distances were computed using the maximum composite likelihood method based on the units of the number of base substitutions per site. The full-length amino acid sequences were then employed to plot a phylogenetic tree based on the neighbor-joining clustering method in MEGA 10.0 (MEGA; https://www.megasoftware.net/; accessed on 22 November 2020). The biosynthetic pathway for flavonoids was mapped to the KEGG database [38].

## 3. Results

### 3.1. Morphology, Full Transcriptome Sequence, and High-Quality Non-Redundant Sequences

**Figure 1:**
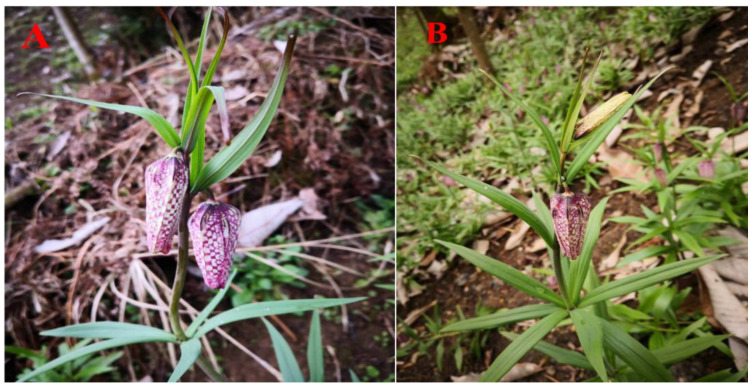
*Fritillaria hupehensis* plant in the field. (**A**) Maturing *F. hupehensis* bulbs and leaves. (**B**) Matured *F. hupehensis* bulbs and leaves.

We sequenced the FL transcriptome of four tissues from *F. hupehensis* plants harvested from Huazhong Medicinal Botanical Garden, China (Figure 1). Morphologically, the plant is 26–50 cm in length, and the bulb is 1.5–3 cm in diameter. The leaves are 3–7-whirled, opposite or scattered centrally, oblong-lanceolate, 7–13 cm long, 1–3 cm wide, and with slightly curved apex. The flowers are usually terminal or axillary, drooping or erect, purple and white colored, bell-shaped with a pedicel of 1–2 cm in length (Figure 1).

**Figure 2:**
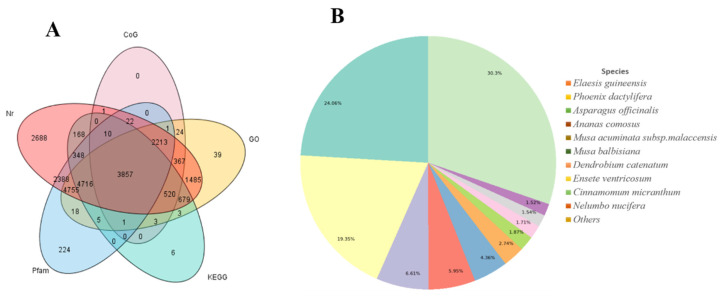
*F. hupehensis* unigenes homology search and isoform detection. (**A**) Venn diagram of number of unigenes with an E-value threshold of 10^−5^ against the protein databases. The numbers in circles indicate the number of individual unigenes annotated by single or multiple databases. (**B**) Percentage of annotated unigenes in Nr database that match the top 11 species using BLASTx.

RNAs were extracted from the bulbs, leaves, roots, and stems (1, 3, and 5 DAA) so that as many transcripts as possible could be identified. The RNAs of the tissues were mixed equally for library preparation to recover more RNA sequences representing the gene expression of the whole plant. Multiple libraries of fractional sizes (1–2, 2–3, and 3–6 k) were created to sequentially transcribe and avoid bias. Five cells (two cells for 1–2 and 2–3 k libraries and one cell for 3–6 k libraries) were used, yielding 342,044 reads. A total of 28,880,638 substrates (clean data) were obtained (Table S1) and filtered with less than 50 bp of subread length and less than 0.75 sequence precision. Each sized library had the expected transcript length distribution ranging from 500 to 4900 bp (Figure 2A,B). A total of 316,438 of the insert reads were FL reads based on bar-coded primers and polyA tails (Table 1). For sequence clustering, the SMRT Analysis (v2.3.0, Pacbio (Menlo Park, CA, USA)) software with the iterative error correction (ICE) algorithm in association with the Quiver program was used. The assembled FL transcriptome was mapped using a genomic mapping and alignment program (GMAP) to provide the highest quality transcripts [47]. Sequences with identities less than 0.9 and coverage less than 0.85 were removed.

Ultimately, 274,919 unique FL non-chimeric (FLNC) transcripts were obtained in *F. hupehensis* (Table 1). We further investigated the alternative splicing (AS) event in the *F. hupehensis* FL transcriptome. In total, 30,961 unigenes experienced AS, with isoform numbers ranging from 1 to 267. The majority of the genes had up to five isoforms, while few genes (*Unigene23270*, *Unigene24755*, *Unigene30347*, *Unigene19170*, *Unigene28276*, *Unigene20453*, *Unigene21361*, *Unigene17114,* and *Unigene28894*) had >100 isoforms and could be a target of further studies to elucidate the impact of AS on their functions (Table S1).

### 3.2. Functional Annotation of Assembled Transcripts

A total of 18,351 of the FLNCs were retrieved using BLASTX (version 2.2.26) and other protein databases including Clusters of Orthologous Groups (COG) [36], Gene Ontology (GO) [37], Kyoto Encyclopedia of Genes and Genomes (KEGG) [38,39], and Protein family (Pfam) [41,42] (Figure 2A). The new isoforms were annotated and their GO functional annotations were used to assign biological processes, cellular components, and molecular terms to *F. hupehensis* unique isoforms. Three primary GO categories and 59 sub-categories were summarized. A large percentage of genes fell under the category “biological process”, “biosynthetic process”, “cellular nitrogen compound metabolism”, and “small molecular metabolic process.” “Cellular component”, “nucleus”, “plastid”, and “cytoplasm” were dominated by the cellular function category; while majority of genes fell under the category of “ion binding”, “molecular function”, “oxidoreductase activity”, and “DNA binding”“ (Figure S1). In the KEGG pathway, the most abundant genes found were associated with the metabolic pathway (2644; 26.0%), followed by the genes for “biosynthesis of secondary metabolites” (1319; 13.0%) and “ribosome” (562; 5.5%), respectively (Table S2). We blasted unigenes in the Nr database using BLASTx and identified 11 species sharing transcriptome similarity with *F. hupehensis* (Figure 2B). *Elaeis guineensis* (Jacq.) (24.06%), *Phoenix dactylifera* (Linn.) (19.35%), *Asparagus officinalis* (Linn.) (6.61%), and *Ananas comosus* (Linn.) Merr. recorded the highest similarity rates (5.59%). These species are important medicinal plants commonly used for their bioactive compounds in folk medicine. This may partly account for the high indices of similarity of their transcripts to that of *F. hupehensis* (Figure 2B). A broad pool of 210 (2.06%) members were mapped to the phenylpropanoid biosynthesis pathway with terpenoid backbone biosynthesis (81%; 0.8%), secondary metabolite biosynthesis pathways, and N-glycan biosynthesis (74%; 0.73%) involved in many transcripts (Table S3). The total annotation of the transcriptome of *F. hupehensis* is a useful resource for studying specific functional processes and important biosynthesis pathways.

### 3.3. Simple Sequence Repeats and Long Non-Coding RNA Analysis

We studied simple sequence repeats (SSRs) and long non-coding RNA (lncRNAs) in the assembled transcripts (Table 2 and Table 3). SSRs have been a common source of genetic markers for mapping, molecular breeding, and population genetic analyses in several species [48]. We used the MicroSAtellite tool (MISA; https://webblast.ipk-gatersleben.de/misa/, accessed on 22 November 2020) to identify SSRs from the unigenes [49]. There were 7,914 SSRs of six types detected, including mono-nucleotides, di-nucleotides, tri-nucleotides, tetra-nucleotides, penta-nucleotides, and hexa-nucleotides (Table S4). Repeat form analyses of the SSRs demonstrated that the most important components (55.7%) were mono-nucleotide SSRs (22.2%), thus confirming earlier transcriptome studies in medicinal plants [48]. Tri-nucleotides (20.7%) were the next most represented class. Only a small proportion of tetra-, penta-, and hexa-nucleotide SSRs (less than 1% each) were found with similar frequencies in the *F. hupehensis* unigenes (Table 2). As identified SSRs are present in the transcript assembly, they could have played roles in *F. hupehensis* gene development [26,50]. The identification of SSRs may significantly improve large-scale genotyping studies, including the assessment of genetic diversity and mapping for different economic traits.

LncRNAs are non-coding RNA transcripts with a length of more than 200 nucleotides found throughout the genome. However, because LncRNA does not code for a protein, transcripts were screened for coding potential to determine if they were LncRNAs. CPC [43], CNCI [44], Pfam [41,42], and CPAT [45] were the four popular coding potential analysis tools used in this study. As non-coding transcripts, a total of 38,607 LncRNAs were found (Table 3).

### 3.4. Candidate Genes involved in Flavonoid Biosynthesis

**Figure 3:**
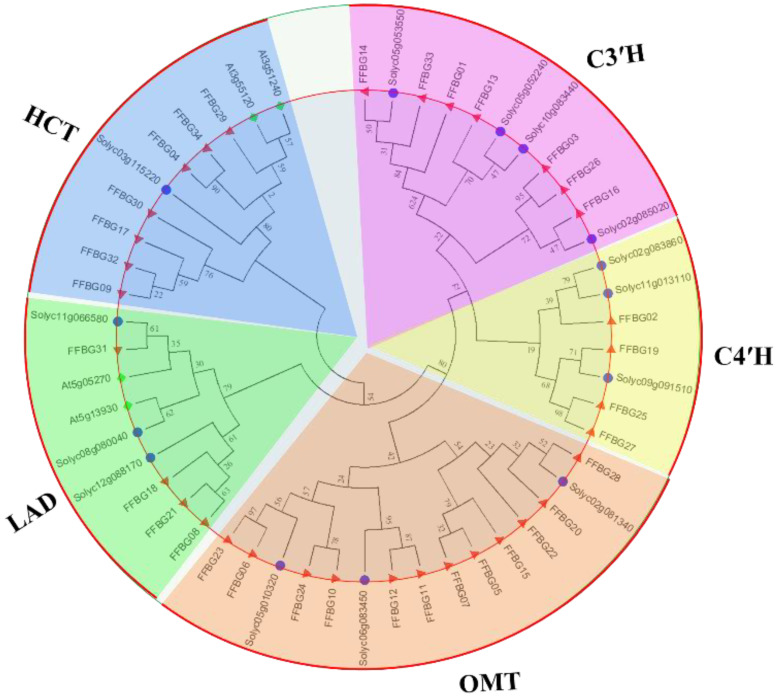
Phylogenetic associations among flavonoid biosynthesis genes in *F. hupehensis* (Hsiao et K.C. Hsia), *Solanum lycopersicum* (Linn), and *Arabidopsis thaliana* (Linn). Purple dot stands for *S. lycopersicum*, green diamond dot is *A. thaliana*, and red dot is *F. hupehensis*. MEGA 10.0 was used to create the phylogenetic tree based on the neighbor-joining method. Clade 1, coumaroylquinate (coumaroyl shikimate) 3′, 4′-monooxygenase genes (C3′H); clade 2 (C4′H); clade 3, O-methyltransferases (OMT); clade 4, ladanein (LAD); and clade 5, shikimate O-hydroxycinnamoyltransferase genes (HCT). On the basis of their clustering patterns, we named the flavonoid biosynthesis genes in *F. hupehensis*.

Flavonoids are the main bioactive compounds in medicinal plants, and their biosynthesis has attracted considerable interest in herbal medicine. In this study, we focused especially on the genes involved in flavonoid biosynthesis. We found 34 unigenes from the KEGG pathway analysis involved in flavonoid biosynthesis (Table S5). For model plants, such as *Arabidopsis thaliana* (Linn) and *Solanum lycopersicum* (Linn), flavonoid biosynthesis is clearly defined; hence, *F. hupehensis* genes from the annotated flavonoid biosynthesis were blasted against flavonoid biosynthesis genes in *S. lycopersicum* and *A. thaliana* (Figure 3). The flavonoid biosynthesis genes in *F. hupehensis* were named based on their clustering patterns (Figure 3). The F. hupehensis flavonoid genes were clustered into five clades: clade 1, coumaroylquinate (coumaroyl shikimate) 3′, 4′-monooxygenase genes (C3′H); clade 2 (C4′H); clade 3, O-methyltransferases (OMT); clade 4, ladanein (LAD); and clade 5, shikimate O-hydroxycinnamoyltransferase genes (HCT). We further annotated the flavonoid biosynthesis genes by analyzing their conserved motifs structure (Figure 3).

## 4. Discussion

Fritillaria (Chinese name Beimu) is a bulbous plant that has long been used as an antitussive and expectorant herb in traditional Chinese medicine [4,51]. Alkaloids, adenisine, flavonoids, saponin, steroids, succinic acid, terpenoids, and thymidine are some of the major phytochemical compounds found in Beimu [4,7,51,52]. For non-model species and non-sequenced genomes, the advent of next-generation sequencing is proving useful in deciphering their potential at the biological, cellular, and molecular levels [11,22]. However, because whole genome sequencing of *F. hupehensis* is not yet available, identifying and comparing gene sequences, discovering new genes and gene biosynthetic pathways, and profiling their expression patterns are somewhat difficult. Due to the lack of a reference genome, molecular studies in Fritallaria has been limiting. Full-length (FL) transcriptome profiling in this important medicinal plant can reveal new information about genes, their expression patterns, and biosynthetic pathways. On the bulbs, leaves, roots, and stems of *F. hupehensis*, we performed FL transcriptome sequencing. In comparison with previous transcriptome studies in *Acacia auriculiformis* (A. Cunn. ex Benth), *Acacia mangium* (Kaneh. & Hatus.) [53], *Eucalyptus grandis* (W. Hill) [54], and *Carthamus tinctorius* (Linn.) [54], we report here more contigs and FL transcripts in *F. hupehensis* [23,29]. This information will add to our understanding of genes in *F. hupehensis* and their functions, which will be useful in future research. Microsatellites (SSRs) are DNA sequences with shorter base pairs (1–6 bp) that have been used as molecular markers because of their co-dominant inheritance, multiallelic nature, reproducibility, relative abundance in genomes, and wider genome coverage [55]. Cultivar identification, genetic relatedness estimation, genome mapping, gene tagging, and germplasm conservation have been undertaken previously using SSRs [56]. The current study discovered genic SSRs that could be part of *F. hupehensis*’ microsatellite repertoire. Their presence in gene transcripts indicates that they are involved in gene expression and functions, in addition to their various forms and roles. They can also be used for gene mapping and population genetic studies to highlight any non-random relationships between markers, genes, or quantitative trait loci (QTLs) in a population [26,55]. The information presented here will be useful for marker-assisted selection to accelerate trait-specific breeding in *F. hupehensis*. Repeat form analyses of the SSRs demonstrated that the most important components (55.7%) were mono-nucleotide SSRs (22.2%), thus confirming their ubiquity as reported by earlier transcriptome studies in medicinal plants [14,21,22,23,26,29].

New groups of LncRNAs, such as promoter-related RNAs and long RNAs, have been discovered as a result of the advancement of modern sequencing technologies [22,57]. Aside from mRNAs, LncRNAs generate a series of transcripts that serve as structural, catalytical, and regulatory RNAs [6,21]. They play a major role in stress response and regulate chromosomal dynamics, RNA editing, splicing, and degradation of mRNAs [57,58]. Plant growth and phenotypic variations have been linked to LncRNA disruption and misexpression [59]. LncRNAs play a critical role in gene regulation and cellular functions such as protein production (rRNA) [22,60]; RNA transcription and post transcription, including splicing [22,60]; and protein processing and cell differentiation [22,60,61,62]. Targeting these LncRNAs is critical for the regulation of crucial cell processes and functions, which will improve *F. hupehensis’* medicinal and pharmaceutical value. 

Most medicinal plants’ therapeutic and pharmaceutical properties are largely determined by their flavonoid profiles. Understanding the biosynthesis and transcriptional regulation of flavonoids in *F. hupehensis* could fast-track the development of this pathway for medicinal and pharmaceutical purposes. Flavonoids have a wide range of structures and colors, and they play important roles in plant metabolism [22]. Except for the flavone biosynthesis gene, we found 34 flavonoid biosynthesis genes in *F. hupehensis*, indicating that this pathway is highly conserved in this *F. hupehensis* (Figure 3 and Figure S2). For instance, clade 1 contains a mix of flavonoid genes from tomato and *F. hupehensis* (Figure 3). The genes contain C3H and C4H cytochrome P450 domain and heme-thiolate proteins reputed for oxidative degradation of phyto-compounds [63]. The C3H and C4H families participate in a series of biosynthetic reactions that lead to the production of phytohormones, secondary metabolites, and lignins [63]. Genes from *F. hupehensis*, tomato, and Arabidopsis were grouped together in clade 3 (OMT family). The OMT family includes O-methyltransferases such as catechol O-methyltransferase and caffeoyl-CoA O-methyltransferase, as well as bacterial O-methyltransferases that may be involved in antibiotic production [64]. The OMT enzyme prefers to methylate flavanones and dihydroflavonols in the para position, whereas flavones and flavonols are methylated in the meta position [65]. It is thought to play a role in the conversion of Caffeoyl-CoA to Feruloyl-CoA in flavonoid biosynthesis. Flavonoid genes from *F. hupehensis* (Hsiao et K.C. Hsia), *S. lycopersicum* (Linn), and *A. thaliana* (Linn) were clustered together in clades 4 (LAD) and 5 (HCT). Several transferase enzymes were found in the HCT family, including anthranilate N-hydroxycinnamoyl/benzoyl transferase [66], which catalyzes the reaction of phytoalexin biosynthesis, and deacetylvindoline 4-O-acetyltransferase (EC:2.3.1.107), which catalyzes the last step in vindoline biosynthesis [67].

Many of the flavonoid biosynthesis genes were found in multi-gene families, implying that *F. hupehensis* has experienced genome duplication. Based on KEGG pathway analysis in *Coptis deltoidei* (Linn.), Lulin et al. [29] identified 156 unigenes as encoding enzymes involved in flavonoid biosynthesis [24]. In the medicinal plant *Carthamus tinctorius* (Linn.), Liu et al. [68] discovered 22 flavonoid-encoding unigenes, including chalcone synthase genes, chalcone isomerase genes, and anthocyanidin synthase genes. We identified candidate transcripts encoding key enzymes involved in flavonoid biosynthesis pathways using the FL transcriptome sequence data, which gave us insights into the bioactive compounds in *F. hupehensis*. However, each family’s isoform that participates in a specific metabolic family needs to be investigated further. Nonetheless, these transcriptome data represent the first genomic resource for *F. hupehensis*, paving the way for future research using biotechnology, genomics, and synthetic biology approaches to improving this ethnomedicinal plant.

## 5. Conclusions

Full-length transcriptome profiling of *F. hupehensis* was performed using the PacBio RS II de novo sequencing method. A total of 342,044 FL transcripts were analyzed, yielding an average transcript length of 1365 bp. The transcripts were functionally annotated and were found to be involved in a number of biological processes. Flavonoid biosynthesis, flavone and flavonol biosynthesis, vitamin B6 metabolism, valine, leucine, and isoleucine biosynthesis, TGF-beta signalling pathway, ubiquinone and other terpenoid-quinone biosynthesis were the most essential biosynthesis pathways uncovered by our KEGG pathway mapping. We also found LncRNAs and SSRs in *F. hupehensis*, which will facilitate further studies on a variety of important cellular functions and gene expression regulation. The genomic-SSRs discovered in this study provide an excellent and cost-effective option for developing functional markers for marker-assisted trait-specific breeding in the species.

## Figures and Tables

**Table 1:** PacBio transcriptome sequencing summary of *F. Hupehensis* tissues.

Library	Number of Reads	Number of Subreads	Number of FL Transcripts	Number of FLNC	Assembly Length (Mb)	Average Transcript Length (bp)	N50 (bp)
Reads	342,044	28,880,638	316,438	274,919	1647	1365	1888

FL and FLNC refer to full-length and full-length non-chimeric transcripts, respectively.

**Table 2:** Profiles of simple sequence repeats (SSRs) detected in *F. hupehensis* transcriptome.

SSR	Number of SSR
Total SSRs	7914
Total SSR length	13387
Relative abundance (SSR/Mb)	143
Relative density (bp/Mb)	243
SSR containing sequences	5973
Sequences containing more than 1 SSR	1311

**Table 3:** Profiles of long non-coding RNA (LncRNAs) identified in *F. hupehensis* transcriptome

LncRNA Length	Number
200–400	1899
400–600	5279
600–800	7986
800–1000	7180
1000–1200	5058
1200–1400	3382
1400–1600	2113
1600–1800	1438
1800–2000	909
2000–2200	723
2200–2400	529
2400–2600	391
2600–2800	314
2800–3000	270
3000–3200	221
3200–3400	185
3400–3600	140
3600–3800	118
3800–4000	96
4000–4200	70
4200–4400	78
4400–4600	49
4600–4800	39
4800–5000	27
5000–5200	26
5200–5400	13
5400–5600	20
5600–5800	15
5800–6000	8
>6000	31

## Data Availability

The complete sequence data have been deposited in the sequence reads archives of NCBI under the project number: PRJNA671629 (https://www.ncbi.nlm.nih.gov/bioproject/?term=PRJNA671629). The transcriptome assembly was also submitted to Transcriptome Shotgun Assembly number the accession number: GJAT00000000.

## Supplementary Materials

The following are available online at https://www.mdpi.com/article/10.3390/life11040287/s1, Figure S1 GO classification of *F. hupehensis* unigenes, Figure S2 KOG functional classification of *F. hupehensis* unigenes, Table S1 Statistic of the isoforms identified per unigene, Table S2 Seven protein databases used for the transcripts annotation, Table S3 Flavonoid-related unigenes and pathways identified in the FL transcriptome, Table S4 Type, motif, and length of SSRs identified, Table S5 Pathways identified from the assembled transcriptome.
